# Drug-Induced Sleep Endoscopy Findings and Hypoglossal Nerve Stimulation Therapy Outcomes

**DOI:** 10.3390/jpm13030532

**Published:** 2023-03-16

**Authors:** Johannes Pordzik, Christopher Seifen, Katharina Ludwig, Berit Hackenberg, Tilman Huppertz, Katharina Bahr-Hamm, Christoph Matthias, Haralampos Gouveris

**Affiliations:** Department of Otolaryngology, Head and Neck Surgery, University Medical Center Mainz, 55131 Mainz, Germany

**Keywords:** sleep endoscopy, tongue base, velar, oropharyngeal, obstructive sleep apnea, hypoglossal nerve stimulation, polysomnography, individualized medicine

## Abstract

Hypoglossal-nerve stimulation (HGNS) is an established second-line therapy for patients with obstructive sleep apnea (OSA). Existing studies investigating the effect of preoperative drug-induced sleep endoscopic (DISE) findings on HGNS outcomes have mainly focused on the apnea/hypopnea index (AHI) among polysomnography (PSG) parameters, and have less frequently tested other PSG parameters such as the apnea index (AI), hypopnea index (HI), oxygen desaturation index (ODI), snoring index, and arousal index, or patient-reported excessive daytime sleepiness. The aim of this study was to investigate the correlation between DISE findings and the above-mentioned metrics after HGNS therapy. We only included patients with DISE findings providing detailed information about the degree of the anteroposterior velar (APV), oropharyngeal lateral wall (OPLW), or tongue-base (BT) obstruction based on the velum, oropharynx, base of tongue, and epiglottis (VOTE) classification. The data of 25 patients (9 female (36%)) were retrospectively evaluated. The mean age at the date of implantation was 54.52 ± 9.61 years, and the mean BMI was 29.99 ± 3.97 kg/m^2^. Spearman’s rho correlation coefficients were calculated. Significant correlations were found between the degree of APV obstruction and postoperative HI (r = −0.5, *p* < 0.05), and between the degree of OPLW obstruction and postoperative snoring index (r = 0.42, *p* < 0.05). BT obstruction was strongly correlated with postoperative metrics such as AHI (r = −0.57, *p* < 0.01), AI (r = −0.5, *p* < 0.05), ODI (r = −0.57, *p* < 0.01), ∆ AHI (r = 0.58, *p* < 0.01), ∆ AI (r = 0.54, *p* < 0.01) and ∆ ODI (r = 0.54, *p* < 0.01). No significant correlation was found between DISE findings and postoperative Epworth Sleepiness Scale values. These findings suggest that preoperative DISE findings, especially the degree of BT obstruction, are important for predicting an HGNS therapy outcome.

## 1. Introduction

The prevalence of obstructive sleep apnea (OSA) is increasing in the general adult population around the globe [1,2]. During sleep, apneas and hypopneas are caused due to intermittent upper-airway collapses [3]. Obstructive sleep apnea is a heterogeneous disorder. There are well-known anatomic (ones related to the passive critical closing pressure of the upper airway (Pcrit)) and nonanatomic (reduced genioglossus muscle responsiveness, low arousal threshold, and reduced respiratory control stability/increased loop gain) contributions to OSA [4]. In addition, the degree of the reduction in the connectivity between the sensorimotor neural output units and the mechanical upper-airway muscular units explains the degree of respiratory disturbance in treatment-naive obstructive sleep apnea patients [5].

Among other negative outcomes, OSA is associated with an increased risk of coronary artery disease [6], hypertension [7], diabetes [8], nonalcoholic fatty liver disease [9], and ischemic cerebrovascular events [10]. Positive airway pressure (PAP) remains the first-line standard therapy for OSA and beneficial. However, many patients do not tolerate PAP therapy. According to some reports, adherence to PAP therapy may be as low as 54%, with some reports claiming 17% adherence when stricter recommendation criteria for daily PAP use are applied [11]. Therefore, many patients and their physicians quite often look for alternatives. Further options for OSA treatment are velar/oropharyngeal soft tissue or maxillomandibular surgery, weight loss, positional therapy (i.e., encouraging side sleeping), and mandibular advancement devices [12]. Over the last decade, respiration-coupled hypoglossal nerve stimulation (HGNS) therapy has been established as a therapeutic alternative for patients not tolerating or failing PAP therapy. Respiration-coupled hypoglossal nerve stimulation therapy had shown a clinically significant reduction in core sleep-related respiratory metrics such as the apnea–hypopnea index (AHI), apnea index (AI), hypopnea index (HI), oxygen-desaturation index (ODI), snoring index, and patient-reported outcome measures (PROMs) such as reduced subjective sleepiness assessed using the Epworth Sleepiness Scale (ESS) [13,14]. Following the current guidelines to treat patients via HGNS, drug-induced sleep endoscopy (DISE) is an indispensable and mandatory component of preoperative evaluation [15]. There are various classification systems with varying degrees of complexity for documenting and comparing DISE findings. The VOTE classification system was established to describe DISE findings focusing on the anatomical structures that cause obstructions, namely, the velum, oropharyngeal lateral wall, tongue base, and epiglottis [16]. The relationship among DISE, AHI, and ESS was previously investigated [17]. Tongue-base collapse was associated with AHI, although no significant correlations were observed between collapses in the lateral, uvulopalatal, and laryngeal zones, and AHI. Furthermore, DISE findings and ESS values were not significantly correlated. These data suggest that preventing tongue-base collapse is very important in treating OSA.

By means of respiration-coupled HGNS therapy. a cuff-based stimulating electrode is implanted to selectively include only hypoglossal nerve branches innervating the protruding and stiffening muscles of the tongue. During surgery, with the use of electrophysiological monitoring, care is taken to exclude the stimulation of hypoglossal nerve branches innervating the retractor muscles of the tongue. Therefore, HGNS therapy should prevent the collapse of the tongue base by promoting a respiration-coupled protrusion of the stiffened tongue. Even if HGNS therapy is an evidence-based therapeutic option, it remains an invasive surgical treatment. Therefore, there is increasing interest in finding relevant preoperative predictors for a positive HGNS therapy outcome. Studies investigating the associations of preoperative DISE findings with HGNS outcomes showed inconsistent results [18,19,20,21,22]. Furthermore, to the best of our knowledge, studies investigating an association between preoperative DISE findings and excessive daytime sleepiness (EDS) after HGNS therapy are lacking. The aim of the present study is to evaluate the impact of preoperative DISE findings and HGNS outcomes such as objective PSG-based sleep parameters and patient-reported outcomes on EDS such as ESS.

## 2. Materials and Methods

We retrospectively assessed patients implanted with a respiration-coupled HGNS using the Inspire Medical System (Maple Grove, MN, USA) between February 2020 and December 2022 in our tertiary care otorhinolaryngology department. We included patients with DISE findings that provided detailed information on the (percentage) degree of the following obstructions based on the velum, oropharynx, base of tongue, and epiglottis (VOTE) classification, focusing on the following [16]: anterior/posterior velar (APV), oropharyngeal lateral-wall (OPLW), and base of the tongue (BT) obstructions. All DISE procedures were performed or supervised by an expert in sleep medicine in our department. Propofol titration was performed by an experienced anesthesiologist according to the current practice guidelines for DISE [23] using a target-controlled infusion (TCI) device under basic cardiorespiratory monitoring such as blood pressure, electrocardiogram, and pulse oximetry. The depth of sedation was monitored continuously during DISE using the bispectral index (BIS) in addition to clinical anesthesiologic assessment. All patients evaluated with DISE in the present analysis fulfilled the current guideline criteria for HGNS therapy [15], such as intolerance to PAP therapy, body mass index (BMI) < 35 kg/m^2^, apnea/hypopnea index (AHI) 15–65 h with <25% central apneas on polysomnography (PSG), the absence of complete velar concentric collapse on drug-induced sleep endoscopy (DISE), and the absence of chronic major psychiatric or neurodegenerative disease. PSG was performed according to the standard American Academy of Sleep Medicine (AASM, Inc.) guidelines [3] before and 102.64 ± 34.36 days after the activation of the HGNS. Polysomnographic recordings involved C3 and C4 EEG recordings, electrooculograms, submental and bilateral pretibial EMGs, and one-lead electrocardiograms. Nasal air flow was detected with the measurement of impact pressure through a nasal sensor that determined the pressure fluctuations of the breathed air stream. Thoracic and abdominal excursions were simultaneously recorded by means of piezoelectric bands, oxyhemoglobin saturation (using a pulse oximeter), and body position. Snoring was recorded with a prelaryngeally fixed microphone. Polysomnographic recordings were performed using the Miniscreen Pro polysomnographic Diagnostic Sleep System (Loewenstein Medical, Bad Ems, Germany). In the morning after, each sleep study night, sleep stage, and sleep-related respiratory event were manually scored according to the American Academy of Sleep Medicine (AASM) guidelines [3]. Nasal air-flow amplitude reduction in airflow signal of 90%, lasting for at least 10 s, was defined as apnea. Hypopnea was defined as a reduction in the airflow signal on the nasal flow sensor between 30% and 90% of the pre-event baseline for 10 s with an associated 3% reduction in arterial blood oxygen saturation (SpO_2_) and/or a cortical arousal. Apnea events were additionally further classified into obstructive, central, or mixed on the basis of the simultaneous visual and manual assessment of nasal air flow, and thoracic and abdominal excursion, performed by an expert in sleep medicine.

The following parameters of the PSG report were investigated: AHI, AI, HI, SI, ODI, and arousal index.

Postoperatively, 17 out of the 25 patients (68%) filled the ESS questionnaire. The questionnaire was given to each patient before the preoperative PSG. Therefore, the patients were directly asked to fill out the questionnaire. The ESS questionnaire was given to every patient during the postoperative PSG, but not every patient completed the questionnaire. Self reported daytime sleepiness was measured by using the german version of the ESS questionnaire [24]. Eight items were rated on a four-point Likert scale. Therefore, an overall score between 0.0 and 24.0 was given. Higher scores represented higher degrees of daytime sleepiness.

Ethics statement: All patients included signed an informed consent form for the use of their data for clinical research. All data were anonymously evaluated. Due to this and the retrospective nature of the study, the local institutional review waived the need for separate approval. This study was conducted in accordance with the Declaration of Helsinki.

### Statistical Analysis

SPSS 27 (IBM, Armonk, NY, USA) was used for statistical analysis. Categorical variables were described as number and percentage (%), continuous variables were described as mean ± standard deviation and ordinal scaled variables were described as median. Comparisons between groups were analyzed according to Wilcoxon’s rank-sum test. Spearman’s correlation coefficient was calculated between two variables. *p* < 0.05 was considered a statistically significant result.

## 3. Results

A total of 25 patients (16 male (64%), 9 female (36%)) aged 54.52 ± 9.61 years at the date of implantation were included. The mean BMI was 29.99 ± 3.97 kg/m^2^. The following PSG-based respiratory metrics were significantly reduced after hypoglossal nerve stimulation (Table 1): AHI, AI, HI, snoring index, ODI (n/hour) (*p* < 0.001, *p* < 0.01, *p* < 0.001 *p* < 0.001, *p* < 0.01, and *p* < 0.01, respectively). The median postoperative ESS score was 14.

The percentage of the degree of obstruction at various anatomical levels is shown in Table 2.

Significant correlations (Spearman’s rho coefficient) were found between the APV obstruction and postoperative HI (r = −0.5, *p* < 0.05), and the obstruction of oropharyngeal lateral wall and postoperative snoring index (r = 0.42, *p* < 0.05). Strong correlations were found between BT obstruction and postoperative PSG metrics such as AHI (r = −0.57, *p* < 0.01), AI (r = −0.5, *p* < 0.05), ODI (r = −0.57, *p* < 0.01), ∆ AHI (r = 0.58, *p* < 0.01), ∆ AI (r = 0.54, *p* < 0.01) and ∆ ODI (r = 0.54, *p* < 0.01) (s. Table 3 and Table 4).

## 4. Discussion

In this study, we demonstrated a strong significant correlation between DISE findings and HGNS therapy success, especially between the degree of obstruction at the BT and postoperative AHI, AI and ODI, and ∆ AHI, ∆ AI, and ∆ ODI. Furthermore, a significant correlation was found between the degree of APV obstruction and postoperative HI, and the degree of the obstruction of the oropharyngeal lateral wall and the postoperative snoring index. No significant correlation was found between any of the DISE findings and postoperative ESS.

HNGS therapy is a well-established second-line therapy for OSA [13,14,25]. HGNS is invasive surgical therapy. Therefore, there is a need to find relevant predictors of the success of postinterventional HGNS therapy. Studies investigating the association of preoperative DISE findings and HGNS outcomes showed inconsistent results [18,19,20,21,22]. One study could prove increased odds for therapy success in patients with complete (vs. partial/no) tongue-related obstruction concerning AHI reduction [21]. A study proved a significant correlation between AHI and the degree of tongue-base collapse during DISE [17]. That study only focused on AHI as a PSG parameter. AI and HI were not subdivided. However, these findings are consistent with our results and rather not surprising, since the selective electrostimulation of specific branches of the hypoglossal nerve and, under favorable anatomic conditions, of the first cervical motor nerve, increases the activity of the genioglossal muscle, the transverse and vertical tongue muscles, and, in the case of the stimulation of the first cervical motor nerve, the geniohyoid muscle. This activation results in the protrusion of the stiffened tongue body [26]. However, our study could prove a significant correlation of obstruction at the BT during DISE and PSG-based metrics that had not been described in this context, such as AI and ODI, and ∆ AHI, ∆ AI, and ∆ ODI.

The degree of APV obstruction is associated with lower postoperative HI, while BT obstruction is associated with lower postoperative AI. This important finding suggests that hypopneas are much more strongly associated with incomplete/nonconcentric velar collapse than with apneas in OSA patients. Another interesting prospective cohort study evaluated the usefulness of using the therapeutic positive airway pressure level applied at the soft palate through a nasal mask interface during DISE as a predictor of the postoperative HGNS outcome. Responders to HGNS therapy had significantly lower mean palatal opening pressure than that of nonresponders, namely, 5.0 vs. 9.2 cm H_2_O, respectively [27]. The same authors found that a palatal opening pressure cut-off level of less than 8 cm H_2_O resulted in a positive predictive power of 82.4% with a concomitant sensitivity of 77.8% and specificity of 66.7%. Nonetheless, their OSA patient cohort included relatively more female patients (48.1%), and showed a much lower mean BMI than that of our cohort (28.1 vs. 30 kg/m^2^). Our cohor, on the other hand, was much younger, with a mean age of 54.5 (vs. 62.0) years. The age of OSA patients has quite important pathophysiologic implications because airway collapsibility plays a relatively greater pathogenic role in older adults, whereas a sensitive ventilatory control system is a more prominent trait in younger adults with obstructive sleep apnea [28]. These parameters should be carefully evaluated and taken into account because they could potentially confound the PAP-associated palatal opening pressure results during DISE [28]. Unfortunately, these authors did not pre- or postoperatively report on the differential contribution of apnea and hypopnea events to the AHI in their cohort. As a result, our visual DISE findings regarding hypopneas and apneas could not be compared. This fact showcases the advantage of providing further detailed information on PSG-related metrics, especially the hypopnea and apnea indices, in future reports regarding DISE and HGNS therapy.

A further report was previously published providing evidence for a 92% positive predictive value for HGNS success in patients when the therapeutic PAP level in PAP-intolerant patients was less than 8 cm H_2_O [29]. Only 44% of PAP-intolerant OSA patients with therapeutic PAP levels greater than or equal to 8 cm H_2_O, on the other hand, exhibited HGNS success [30].

Upper-airway collapsibility is a key determinant of obstructive sleep apnea that can influence the efficacy of noncontinuous positive airway pressure treatments for OSA [30]. Upper-airway collapsibility is characterized by passive pharyngeal critical closing pressure (Pcrit). Landry et al. found that a therapeutic CPAP level ≤ 8.0 cm H_2_O was sensitive (89%) and specific (84%) in detecting a mildly collapsible (namely, with a Pcrit ≤ −2 cmH_2_O) upper airway. Therefore, according to these authors [30], a patient’s therapeutic CPAP requirement has a strong predictive relationship with their Pcrit and may be used to accurately differentiate obstructive sleep apnea patients with mild airway collapsibility from those with moderate-to-severe collapsibility. Due to the equivalent results between the airflow-based and visual assessments of pharyngeal opening pressures during DISE, the visual assessment of pharyngeal opening pressure should be considered a standardized objective parameter in clinical DISE [31].

Due to the existence of palatoglossal mechanical coupling [32], HGNS therapy may cause a significant reduction in hypopnea in patients with significant APV (although nonconcentric) collapse on DISE. This is a very interesting observation that should be further investigated. If this finding could be replicated in future studies, OSA patients with a significant proportion of hypopneas among the total number of their respiratory events during sleep and with a considerable APV obstruction on DISE would particularly profit from inspiratory-coupled HGNS therapy. This could become a major predictor of HGNS therapy outcome in such a selected subgroup of OSA patients. There are inconsistent findings concerning HGNS therapy response and the degree of velar obstructions on DISE. One study showed fewer preoperative velar obstructions associated with a good therapy response [22], in contrast to a study that could not find any clear association between response rates and the velum-related degree or configuration of the obstruction [21]. However, both studies only focused on the AHI as a variable. Our findings suggest that further PSG-based metrics should be considered in future studies of this kind, and further investigated as predictors of HGNS therapy outcome related to preoperative DISE findings.

Postoperatively, the degree of obstruction at the OPLW level was significantly positively correlated with the snoring index. The degree of the collapse of the upper airway, especially at the base of the tongue and the lateral pharynx wall, correlated significantly with the severity of OSA [33,34]. Furthermore, higher snoring frequency and intensity were significantly correlated with obstruction at the OPLW in patients undergoing DISE as an alternative therapy to PAP [35]. However, another study showed OPLW obstruction associated with poorer HGNS outcomes, explaining this result by means of the glossopharyngeal coupling being responsible for the movement of the OPLW [21]. Therefore, this finding suggests that HGNS may only very weakly and rather indirectly affect the movement of the lateral oropharyngeal walls. These indirect forces seem to be too weakly affected by HGNS therapy to reduce AHI, AI, HI, or ODI. Any significant obstruction at the OPLW level may, in some not yet well-defined way, promote snoring while treating apneas and hypopneas in patients who are treated with HGNS therapy.

When interpreting the findings of DISE, one should be very cautious because of the differential effect of sleep onset during DISE on tensor palatini and genioglossus muscles. Upper-airway muscles such as genioglossus and tensor palatini show reduced activity at sleep onset. The reduced muscular activity of the genioglossus is primarily due to inspiratory modulated motor units becoming silent, suggesting reduced respiratory pattern generator output [36]. A greater proportion of expiratory modulated motor units are active in the tensor palatini muscle and, along with inspiratory units, tend to become silent over sleep onset. Therefore, both expiratory and inspiratory drive components from the respiratory pattern generator are reduced at sleep onset in the tensor palatine muscle [36]. These physiological properties may influence the pattern of motion of the soft palate (tensor palatini muscle) and/or of the base of the tongue (genioglossal muscle) during the sleep onset time window, which actually overlaps with the few minutes of the DISE study.

No significant correlation was found between DISE findings and postoperative ESS. Therefore, daytime sleepiness did not seem to be directly affected by upper-airway mechanics and/or anatomical findings in OSA patients. Some authors were able to prove a correlation between sleepiness and AHI [37,38,39]. However, further statements based on an international consensus [40] and the majority of published papers [17,41,42,43,44] suggest that excessive daytime sleepiness is not clearly or linearly associated with AHI. One of those studies investigated the relationship among DISE, AHI, and ESS [17]. ESS seems to be strongly affected by personal structure. From a pathophysiological neurophysiologic point of view and on the basis of emerging evidence, brain activity at the sensorimotor cortex of OSA patients may capture some of the features that are associated with excessive daytime sleepiness in these patients [45]. In accordance with such evidence, a correlation between the ESS score and several questionnaires testing for depression was observed [43].

The limitations of our study are its retrospective nature and relatively small sample. Many previous studies showed HGNS therapy to be efficient and safe [13,46,47]. A further limitation of the present study is the relatively short-term follow-up (i.e., 3 months on average). The postoperative AHI was 22.56 per hour of sleep as shown in Table 1. This is equal to a reduction of approximately 37% in AHI. The AHI reduction was low compared to most previous published reductions. A significant reduction of 52% in the AHI after 12 months in 124 patients was reported by the STAR Trial [13]. A 67% AHI reduction from 33.8 (15.5) to 11.0 (13.6) events/h was demonstrated in a pooled analysis of 584 patients in different international studies such as the STAR trial, a German cohort, a US cohort, and the ADHERE Registry (international registry: data on adherence and outcome of upper airway stimulation) [47]. A significant reduction of 82% in AHI is the highest reported [25]. Therefore, data from our study represent a short-term outcome, namely, 3 months after the activation of the HGNS implant. Further improvement in the AHI with the further continuation of the titration of the device’s neurostimulation parameters in a long-term follow-up is expected. This is mainly caused by the difficult process of optimizing the HGNS stimulation level within the range of 0.1 to 5.0 V. This process may need further appointments and thereby last for months.

Other studies investigated the association between DISE and postoperative HGNS therapy outcome. Nonetheless, they mainly focused on the AHI as the variable of interest [21,22] and excluded other rather significant PSG parameters such as the apnea, hypopnea, oxygen-desaturation, snoring, and arousal indices, and patient-reported excessive daytime sleepiness. Notably, one study investigated the relationship among DISE, AHI, and ESS, but not for patients treated with HGNS therapy [17].

## 5. Conclusions

We demonstrated a strong significant correlation between DISE findings and HGNS therapy outcomes. A higher degree of tongue-base obstruction was significantly associated with lower postoperative AHI, AI, and ODI, and higher reductions in AHI, AI, and ODI. Furthermore, a significant correlation was found between the degree of anteroposterior velar obstruction and postoperative HI, and the degree of the obstruction of the oropharyngeal lateral wall and the postoperative snoring index. These preliminary findings suggest that preoperative DISE findings, especially the degree of obstruction at the base of the tongue, are important predictors of HGNS therapy outcome. In addition, we provided evidence that a high degree of anteroposterior velar nonconcentric obstruction in OSA patients with a significant proportion of hypopnea events may be a significant predictor of a positive HGNS outcome.

## Figures and Tables

**Table 1:** Pre- and postoperative respiratory PSG-based parameters.

	Preoperative	Postoperative	Comparison (*p*-Value)
AHI (n/hour)	35.58 ± 12.32	22.56 ± 13.96	<0.001 (0.0001)
Apnea index (n/hour)	13.54 ± 11.8	8.69 ± 11.17	<0.01 (0.0077)
Hypopnea index (n/hour)	22.05 ± 8.32	13.88 ± 8.32	<0.001 (0.0006)
Snoring index (n/hour)	244.40 ± 156.5	121.98 ± 130.17	<0.001 (0.00002)
Oxygen desaturation index (n/hour)	33.77 ± 15.29	27.05 ± 17.13	<0.01 (0.0084)
Arousal index (n/hour)	18.04 ± 7.69	12.82 ± 7.15	<0.01 (0.0071)

**Table 2:** Preoperative DISE findings (mean ± standard deviation).

	Percentage of Obstruction (%)
Anteroposterior velar obstruction level	84.08 ± 15.76
Oropharyngeal lateral-wall obstruction level	16.80 ± 17.43
Tongue-base obstruction level	54.83 ± 17.14

**Table 3:** Spearman’s rho (r) correlation coefficients for DISE findings with various postoperative PSG metrics and ESS score.

		AHI Post	AI Post	HI Post	ODI Post	Snoring Index Post	Arousal Index Post	ESS Post
Anteroposterior velar obstruction	r	−0.29	−0.01	−0.5	−0.28	−0.11	−0.37	−0.31
	*p*-value	0.15	0.96	<0.05 (0.01)	0.17	0.59	0.07	0.23
Oropharyngeal lateral-wall obstruction	r	0.33	0.25	0.14	0.32	0.42	0.21	0.19
	*p*-value	0.11	0.23	0.51	0.11	<0.05 (0.0358)	0.3	0.46
Tongue-base obstruction	r	−0.57	−0.5	−0.2	−0.57	−0.04	−0.11	−0.13
	*p*-value	<0.01 (0.0027)	<0.05 (0.0107)	0.35	<0.01 (0.0029)	0.86	0.61	0.61

**Table 4:** Spearman’s rho (r) correlation coefficients for DISE findings with various differences (∆) in pre- to postoperative PSG metrics and ESS.

		∆ AHI	∆ AI	∆ HI	∆ ODI	∆ Snoring Index	∆ Arousal Index	∆ ESS
Anteroposterior velar obstruction	r	0.04	0.14	0.13	−0.1	−0.17	0.13	0.21
	*p*-value	0.84	0.52	0.54	0.64	0.42	0.55	0.41
Oropharyngeal lateral-wall obstruction	r	0.04	−0.19	0.32	0.03	0.3	−0.18	−0.16
	*p*-value	0.85	0.37	0.12	0.87	0.15	0.39	0.54
Tongue-base obstruction	r	0.58	0.54	0.2	0.54	−0.12	0.35	−0.42
	*p*-value	<0.01 (0.0025)	<0.01 (0.0052)	0.34	<0.01 (0.0049)	0.56	0.09	0.1

## Data Availability

Please contact the authors for data requests.
